# Dynamics of the functions $f_\mu (z)=z\exp (z+\mu )$ with the real parameter

**DOI:** 10.1186/s40064-016-2411-2

**Published:** 2016-06-23

**Authors:** Xiaocheng Deng, Fanning Meng, Jianming Lin, Wenjun Yuan

**Affiliations:** School of Mathematics and Information Science, Key Laboratory of Mathematics and Interdisciplinary Sciences of Guangdong Higher Education Institutes, Guangzhou University, Guangzhou, 510006 People’s Republic of China; School of Economic and Management, Guangzhou University of Chinese Medicine, Guangzhou, 510006 People’s Republic of China

**Keywords:** Julia set, Fatou set, Periodic point, Critical value, Primary 37F10, Secondary 30D05

## Abstract

In this paper, the dynamics of the functions $f_{\mu }(z)=z\exp (z+\mu )$ with the real parameter is studied. We say that a real parameter $\mu$ belongs to the set $B_n$ for a positive integer *n* if $f_\mu$ has an attracting cycle of *n*-order. We prove that the Fatou set $F(f_\mu )$ is a completely invariant attracting basin for every parameter $\mu <0$. Further, regarding the set $B_n$ for $n>1$, we prove the following results: (1) There exists $\mu _{*}\ne +\infty$ such that $B_2 = (2,\mu _{*})$. (2) For every positive integer $n>2$, the set $B_n$ is non-empty. (3) For every prime number $p>3$, the set $B_p$ has at least two components.

## Introduction and main results

Let $f^n$ be the *n*-th iterate of a transcendental entire function *f*. The maximal open set *F*(*f*) where the family $\{f^n\}_{n=0}^{\infty }$ is normal in the sense of Montel is called the Fatou set, and its complement $J(f):={\mathbb {C}}\backslash F(f)$ is called the Julia set. The dynamics given by the iteration of transcendental entire maps has been widely studied (cf. Eremenko and Lyubich 1992).

Baker (1970) first obtained an entire function *f* with the property $J(f)={\mathbb {C}}$. He proved the following Theorem.

### **Theorem 1**

*For a certain real positive valuek* , *the function*$f(z)=kze^z$*has the whole plane for its setJ*(*f*).

After that, many authors (cf. Fagella 1995; Jang 1992; Kuroda and Jang 1997; Morosawa 1998) studied the dynamics of the functions $f_{\mu }(z):=z\exp (z+\mu )$. Jang (1992) proved that the set

$$\begin{aligned} B_0:=\{\mu \in \mathbb {R} | J(f_\mu )={\mathbb {C}}\} \end{aligned}$$

is an infinite set. Further, Morosawa (1998) proved that the one-dimensional Lebesgue measure of $B_0$ is positive.

The function $f_{\mu }$ has only two singular values: an asymptotic value 0 and a critical value $f_{\mu }(-1)$, hence the Fatou set $F(f_\mu )$ has no wandering components. The asymptotic value is fixed, hence there is only one free singular orbit. It follows that there is at most one cycle of periodic Fatou components, either attracting, parabolic or Siegel. Since for real parameters the orbit of the free critical value is entirely real, there is no possibility of Siegel discs. Hence only attracting or parabolic cycles are possible and attracting or parabolic periodic points (if they exist) are real.

In this paper, our main goal is to study the structure of $B_n$, where

$$\begin{aligned} B_n:=\{\mu \in {\mathbb {R}} | f_\mu \text{ has } \text{ a } \text{ cycle } \text{ of } \text{ attracting } \text{ periodic } \text{ points } \text{ of } n\hbox {-order} \}, \end{aligned}$$

for every positive integer *n*.

For every real parameter $\mu$, $f_\mu$ has two real fixed points 0 and $-\mu$. The multiplier of 0 is $e^{\mu }$, and the multiplier of $-\mu$ is $1-\mu$. Hence $\mu \in {B_1}$ if and only if $\mu$ satisfies the following condition:

$$\begin{aligned} -1<e^{\mu }< 1 \quad \text{or} \quad -1<1-\mu < 1. \end{aligned}$$

This immediately implies that $B_1=(-\infty ,0)\cup (0,2)$.

Since a completely invariant domain contains all singular values, it is easy to see that if $\mu \in (0, 2)$, then the Fatou set $F(f_\mu )$ is not a completely invariant attracting basin. However, for $\mu \in (-\infty ,0)$, we have the following result.

### **Theorem 2**

*For every parameter*$\mu <0$, *the Fatou set*$F(f_\mu )$*is a completely invariant attracting basin.*

Regarding the set $B_n$ for $n>1$, we prove the following Theorems.

### **Theorem 3**

*There exists*$\mu _{*}\ne +\infty$*such that*$B_2 = (2,\mu _{*})$.

### **Theorem 4**

*For every positive integer*$n>2$, $B_n \ne \emptyset$.

### **Theorem 5**

*For every prime number*$p>3$*, the set*$B_p$*has at least two components.*

### *Remark 6*

We believe that $B_3$ is also an interval and Theorem 5 holds also for every integer $n>3$. An interesting problem is how many components contained in $B_p$.

## The Proof of Theorem 2

In order to prove Theorem 2, we need the following Lemmas. Set $h_r(x):=r^2\exp (-2x)-x^2$ and $\Delta _r:=\{z\in {\mathbb {C}}|\ |z|<r\}$.

### **Lemma 7**

*Let*$r\in (0, e^{-1})$, *then*$h_r$*has 3 distinct zeros*$x_1<-1, x_2\in (-1, 0)$*and*$x_3>0$. *Moreover, the solving set of inequality*$h_r(x)\ge 0$*is the union of*$I_1=(-\infty , x_1]$*and*$I_2=[x_2, x_3]$.

### *Proof*

Noting $f_0(x)=xe^x$ and $h_r(x)=e^{-2x}(r^2-x^2e^{2x})$, we have

1 $$\begin{aligned} h_r(x)=0 \Leftrightarrow |f_0(x)|=|r|, \end{aligned}$$

and

2 $$\begin{aligned} h_r(x)>0 \Leftrightarrow |f_0(x)|<|r|. \end{aligned}$$

From $f'_0(x)=(x+1)e^x$, we see that $f_0(x)$ is decreasing in $(-\infty , -1]$ and increasing in $[-1, +\infty )$, and $f_0(-1)=-e^{-1}$ is the minimum value of $f_0(x)$. Note that

$$\begin{aligned} f_0(0)=0, \ \ \lim _{x\rightarrow -\infty }f_0(x)=0 \ \ \text{ and }\ \ \lim _{x\rightarrow +\infty }f_0(x)=+\infty , \end{aligned}$$

if $r\in (0, e^{-1})$, then we infer that $f_0(x)=r$ has the only one root $x_3>0$, and $f_0(x)=-r$ has two roots $x_1<-1$ and $x_2\in (-1, 0)$. Moreover, the solving set of inequality $|f_0(x)|<|r|$ is the union of $(-\infty , x_1)$ and $(x_2, x_3)$. Hence from (1) and (2), we obtain the assertion. $\square$

### **Lemma 8**

*Let*$r\in (0, e^{-1})$, *then*$f_0^{-1}(\Delta _r)$*has two connected components*$D_1$*and*$D_2$, *and the set*$D_1\cup D_2\cup (-\infty , 0)$*is connected.*

### *Proof*

For every $z=x+iy\in {f_0^{-1}(\Delta _r)}$, we have

$$\begin{aligned} |f_0(z)|=|z\exp (z)|<r, \end{aligned}$$

which implies

$$\begin{aligned} \sqrt{x^2+y^2}\exp (x)<r. \end{aligned}$$

It follows that

$$\begin{aligned} |y|<\sqrt{h_r(x)}. \end{aligned}$$

From Lemma 7, we know that the graph of $|y|=\sqrt{h_r(x)}$ consists of two curves

$$\begin{aligned} L_1:\ |y|=\sqrt{h_r(x)},\ \ x\in {I_1} \end{aligned}$$

and

$$\begin{aligned} L_2:\ |y|=\sqrt{h_r(x)},\ \ x\in {I_2}. \end{aligned}$$

Therefore, $f_0^{-1}(\Delta _r)$ has two connected components $D_1$ and $D_2$, where $\partial {D_1}=L_1$ and $\partial {D_2}=L_2$. Obviously the set $D_1\cup D_2\cup (-\infty , 0)$ is connected. Hence we obtain the assertion. $\square$

### **Lemma 9**

*Let*$I=(a,b)$*be an open interval, and*$f: I \rightarrow I$*be a continuous mapping.*

1. If $f(x)>x$ for every $x\in I$, then we have
  $$\begin{aligned} \lim _{n\rightarrow +\infty } f^n(x)=b. \end{aligned}$$
2. If $f(x)<x$ for every $x\in I$, then we have
  $$\begin{aligned} \lim _{n\rightarrow +\infty } f^n(x)=a. \end{aligned}$$

### *Proof*

(1) Suppose $f(x)>x$ for every $x\in I$. Then it follows that the sequence $\{f^n(x)\}_{n=1}^{\infty }$ is increasing. Hence the sequence $\{f^n(x)\}_{n=1}^{\infty }$ either tends to $+\infty$ or tends to $x_0<+\infty$. If the first case happens, then we have $b=+\infty$. If the second case happens, then we infer $x_0=b$. Otherwise, $x_0<b$, and then $x_0$ is a fixed point of *f*, which contradicts that $f(x)>x$ for every $x\in I$. Thus, we obtain that the sequence $\{f^n(x)\}_{n=1}^{\infty }$ tends to *b*.

(2) Similar as the proof of (1), we can obtain (2) easily. $\square$

**Proof of Theorem**2

### *Proof*

Let $\mu <0$. Then singular value 0 of $f_\mu$ is an attracting fixed point. Denote the immediate attracting basin of 0 by *D*. For every $x<0$, we have

$$\begin{aligned} 0>f_\mu (x)=x\exp (x+\mu )>x, \end{aligned}$$

from Lemma 9, it follows that $\lim _{n\rightarrow {+\infty }}f_\mu ^n(x)=0$. Hence $x\in F(f_\mu )$, and then $(-\infty , 0)\subset D$.

Take *r* enough small such that $r_1:=re^{\mu }<-\mu$ and $r<e^{-1}$. Then $r_1+\mu <0$. For every $z\in \Delta _{r_1}$, we have

$$\begin{aligned} |f_\mu (z)|=|z\exp (z+\mu )|\le r_1\exp (r_1+\mu )<r_1. \end{aligned}$$

This implies $\Delta _{r_1}\subset D$. Hence $f_\mu ^{-1}(\Delta _{r_1})\subset F(f_\mu )$.

It is easy to see that $|f_\mu (z)|<r_1\Leftrightarrow |f_0(z)|<r,$ which implies $f_\mu ^{-1}(\Delta _{r_1})=f_0^{-1}(\Delta _r)$. From Lemma 8, we know that $f_\mu ^{-1}(\Delta _{r_1})$ has two connected components $D_1$ and $D_2$, the set $D_1\cup D_2\cup (-\infty , 0)$ is connected. Since $(-\infty , 0)\subset D$, we infer $f_\mu ^{-1}(\Delta _{r_1})\subset D$. Hence *D* is completely invariant. Since the Fatou set $F(f_\mu )$ has at most one cycle of periodic components, and has no wandering components, we have $F(f_\mu )=D$. Thus, Theorem 2 is proved completely. $\square$

##  Dynamics of $f_\mu (x)$ for $\mu \le 2$ and the Proof of Theorem 3

For a real parameter $\mu$, the attracting periodic points of $f_\mu$ (if they exist) are real. From now on, we suppose that the function $f_\mu$ only defined in $\mathbb {R}$.

It is known that $f_\mu$ has only two fixed points 0 and $-\mu$, the multiplier of 0 is $e^{\mu }$, and the multiplier of $-\mu$ is $1-\mu$. We see that the periodic point 0 of $f_\mu$ is attracting (resp. parabolic) for $\mu <0$ (resp. $\mu =0$), the fixed point $-\mu$ of $f_\mu$ is attracting for $\mu \in (0,2)$, and the fixed point $-\mu =-2$ of $f^2_\mu$ is parabolic for $\mu =2$. So the behavior of the iteration of $f_\mu$ for $\mu \le 2$ should be simple. Indeed, we have the following result.

### **Theorem 10**

1. *If*$\mu \le 0$, *then every point in*$(-\infty , -\mu )$*is absorbed by the fixed point* 0 *and every point in*$(-\mu , +\infty )$*escapes to*$+\infty$*under iteration of*$f_\mu$.
2. *If*$\mu \in (0, 2]$, *then every point in*$(-\infty , 0)$*is absorbed by the fixed point*$-\mu$*and every point in*$(0, +\infty )$*escapes to*$+\infty$*under iteration of*$f_\mu$.

Before proving Theorem 10, we first introduce some preliminary facts.

For the function $f_\mu$, we have $f'_\mu (x)=(x+1)\exp (x+\mu )$, it follows that $f_\mu$ is decreasing in $(-\infty , -1]$ and increasing in $[-1, +\infty )$, and $s:=f_\mu (-1)$ is the minimum value of $f_\mu$. Moreover, we see that the following Claims hold.

### **Claim 1**

*If*$$ \mu \le 0 $$*, then*

1. $$f_\mu (x)>x, \ \ {for\;every }\quad x\in (-\mu , +\infty )$$;
2. $$0<f_\mu (x)<x, \ \ {for\;every }\quad x\in (0, -\mu ),\ \ \mu \ne 0$$;
3. $$0>f_\mu (x)>x, \ \ {for\;every }\quad x\in (-\infty ,0)$$.

### **Claim 2**

*If*$$ \mu > 0 $$*, then*

1. $$f_\mu (x)>x, \ \ {for\;every }\quad x\in (0, +\infty )$$;
2. $$f_\mu (x)<x, \ \ {for\;every }\quad x\in (-\mu , 0)$$;
3. $$f_\mu (x)>x, \ \ {for\;every }\quad x\in (-\infty , -\mu )$$.

Since $f_\mu (x)$ is increasing in $[-1, +\infty )$ and $f_\mu (-\mu )=-\mu$, by Claim 2, we obtain the following Claim.

### **Claim 3**

*If*$$ \mu \in (0, 1] $$*, then*

1. $$x>f_\mu (x)>-\mu , \ \ \ {for\;every }\quad x\in (-\mu , 0)$$;
2. $$x<f_\mu (x)<-\mu , \ \ \ {for\;every }\quad x\in (-1, -\mu ),\ \ \mu \ne 1$$.

Let $$ g_\mu (x):=x+f_\mu (x)+2\mu $$. Then

3 $$\begin{aligned} f^2_\mu (x) =f_\mu (x)\exp (f_\mu (x)+\mu ) =x\exp (x+\mu )\exp (f_\mu (x)+\mu ) =x\exp (g_\mu (x)). \end{aligned}$$

For the function $g_\mu (x)$, we have

4 $$\begin{aligned} g'_\mu (x)=1+(x+1)\exp (x+\mu ) \end{aligned}$$

and

5 $$\begin{aligned} g''_\mu (x)=(x+2)\exp (x+\mu ). \end{aligned}$$

From (5), we see that the curve $y=g_\mu (x)$ is convex in $(-\infty , -2]$ and concave in $[-2, +\infty )$. Furthermore, we have the following two Lemmas.

### **Lemma 11**

*If*$\mu \le 2$, *then the function*$g_\mu (x)$*is increasing in*$(-\infty , +\infty )$.

### *Proof*

From (4), we have

$$\begin{aligned} g'_\mu (-2)=1-\exp (\mu -2)\ge 0. \end{aligned}$$

Since the curve $y=g_\mu (x)$ is convex in $(-\infty , -2]$ and concave in $[-2, +\infty )$, we infer that the function $g_\mu (x)$ is increasing in $(-\infty , +\infty )$. $\square$

### **Lemma 12**

*If*$\mu >2$, *then the function*$g_\mu$*has only three distinct zeros*$-\mu , p$*andq* , *where*

6 $$\begin{aligned} p<-\mu \ \ \ \ \text{ and } \ \ \ \ -2<q<0, \end{aligned}$$

*moreover*

7 $$\begin{aligned} g'_\mu (p)>0 \ \ \ \ \text{ and } \ \ \ \ g'_\mu (q)>0. \end{aligned}$$

### *Proof*

From (4), we have

$$\begin{aligned} g'_\mu (-\mu )=1+(-\mu +1)\exp (-\mu +\mu )=2-\mu <0. \end{aligned}$$

Note that $g_\mu (0)=2\mu >0$ and $g_\mu (-\mu )=0$. Since the curve $y=g_\mu (x)$ is convex in $(-\infty , -2]$ and concave in $[-2, +\infty )$, we infer that the function $g_\mu$ has only three distinct zeros $-\mu , p$ and *q* , where $p<-\mu$ and $-2<q<0,$ moreover, $g'_\mu (p)>0$ and $g'_\mu (q)>0.$

**Proof of Theorem**10

### *Proof*

First, we prove the part (1) of Theorem 10.

From Claim 1, by Lemma 9, we infer that for $\mu \le 0$,

$$\begin{aligned} \lim _{n\rightarrow +\infty } f^{n}_\mu (x)=+\infty , \ \ \ \text{ for } \text{ every } \; x\in (-\mu , +\infty ), \end{aligned}$$

and

$$\begin{aligned} \lim _{n\rightarrow +\infty } f^{n}_\mu (x)=0, \ \ \ \text{ for } \text{ every } \; x\in (-\infty , -\mu ), \end{aligned}$$

i.e., every point in $(-\mu , +\infty )$ escapes to $+\infty$ and every point in $(-\infty , -\mu )$ is absorbed by the fixed point 0 under iteration of $f_\mu$. Thus, the part (1) of Theorem 10 is proved.

Next, we prove the part (2) of Theorem 10.

From Claim 2, by Lemma 9, we infer that every point in $(0, +\infty )$ escapes to $+\infty$ under iteration of $f_\mu$ for $\mu > 0$.

For every $x\in (-\infty , 0)$, we have $f_\mu (x)\in [s, 0)$. Hence, once we have proven that every point in [*s*, 0) is absorbed by the fixed point $-\mu$ under iteration of $f_\mu$, then it follows that every point in $(-\infty ,0)$ is also absorbed by the fixed point $-\mu$ under iteration of $f_\mu$.

Note that $s>-1$ for $\mu \in (0, 1)$ and $s=-1$ for $\mu =1$. Using Lemma 9, from Claim 3, we infer that for $\mu \in (0, 1]$, every point in [*s*, 0) is absorbed by the fixed point $-\mu$ under iteration of $f_\mu$, and then every point in $(-\infty , 0)$ is also absorbed by the fixed point $-\mu$ under iteration of $f_\mu$.

The remainder to be proved is the following claim:

*For*$\mu \in (1,2]$, *every point in* [*s*, 0) *is absorbed by the fixed point*$-\mu$*under iteration of*$f_\mu$.

Suppose $\mu \in (1, 2]$. By Lemma 11, $g_\mu (x)$ is increasing in $(-\infty , +\infty )$, we have

$$\begin{aligned} g_\mu (x)>g_\mu (-\mu )=0, \ \ \ \text{ for } \text{ every } \; x\in (-\mu , 0). \end{aligned}$$

Hence by (3), we have

8 $$\begin{aligned} f^2_\mu (x)=x\exp (g_\mu (x))<x, \ \ \ \text{ for } \text{ every } \; x\in (-\mu , 0). \end{aligned}$$

Since $f_\mu (x)$ is decreasing in $(-\infty ,-1]$, noting $f_\mu (-\mu )=-\mu$ and $f_\mu (s)=f^2_\mu (-1)<-1$, we have

9 $$\begin{aligned} s< f_\mu (x)<-\mu , \ \ \ \text{ for } \text{ every } \; x\in (-\mu ,-1) \end{aligned}$$

and

10 $$\begin{aligned} -\mu<f_\mu (x)<-1,\ \ \ \text{ for } \text{ every } \; x\in [s, -\mu ). \end{aligned}$$

From (8), (9) and (10), we obtain

$$\begin{aligned} -\mu<f^2_\mu (x)<x,\ \ \ \text{ for } \text{ every } \; x\in (-\mu ,-1). \end{aligned}$$

Hence by Lemma 9, we get

$$\begin{aligned} \lim _{n\rightarrow +\infty } f^{2n}_\mu (x)=-\mu , \ \ \ \text{ for } \text{ every } \; x\in (-\mu ,-1), \end{aligned}$$

and then

$$\begin{aligned} \lim _{n\rightarrow +\infty } f^{2n+1}_\mu (x)=f_\mu (-\mu )=-\mu , \ \ \ \text{ for } \text{ every } \; x\in (-\mu ,-1). \end{aligned}$$

Thus we obtain

$$\begin{aligned} \lim _{n\rightarrow +\infty } f^{n}_\mu (x)=-\mu , \ \ \ \text{ for } \text{ every } \; x\in [-\mu ,-1). \end{aligned}$$

Further, from (10), we have

11 $$\begin{aligned} \lim _{n\rightarrow +\infty } f^{n}_\mu (x)=-\mu , \ \ \ \text{ for } \text{ every } \; x\in [s,-1). \end{aligned}$$

i.e., every point in $$ [s,-1) $$ is absorbed by the fixed point $$ -\mu $$ under iteration of $$ f_\mu $$. For every $$ x\in [-1,0) $$, we have

$$\begin{aligned} f_\mu (x)=x\exp (x+\mu )<x. \end{aligned}$$

Assume $f^n_\mu (x)\ge -1$ hold for all positive integer *n*. Then it follows that the sequence $\{f^n_\mu (x)\}_{n=1}^{\infty }$ is decreasing, hence it tends to a fixed point $x_0\in [-1,0)$ of $f_\mu$. This contradicts that $f_\mu (x)=x\exp (x+\mu )<x$ for every $x\in [-1,0)$. So there exists a positive integer *k* such that $f^k_\mu (x)\in [s, -1)$, and it follows from (11) that

$$\begin{aligned} \lim _{n\rightarrow +\infty } f^{n}(f^k_\mu (x))=-\mu , \end{aligned}$$

which implies that *x* is absorbed by the fixed point $-\mu$ under iteration of $f_\mu$.

Thus, we completed the proof of Theorem 10. $\square$

As a corollary of Theorem 10, we have the following result.

### **Theorem 13**

*If*$\mu \le 2$, *then*$f_\mu$*has no periodic points ofn* -*order for any*$n \ge 2$.

From (3) and Lemma 12, we immediately get the following result.

### **Lemma 14**

*For every*$\mu >2$, $f_\mu$*has only one cycle of periodic points of 2-order.*

Let $\{p, q\}$ be the cycle of periodic points of 2-order of $f_\mu$ for $\mu >2$, which satisfies (6) and (7). Note that $g_\mu (p)=0$ and $q=f_\mu (p)=p\exp (p+\mu )$, which imply

12 $$\begin{aligned} p+q+2\mu =0, \end{aligned}$$

then from (4), we have

13 $$\begin{aligned} g'_\mu (p)=1+(p+1)\exp (p+\mu )=1+\frac{q(p+1)}{p}=\frac{p+q+pq}{p}. \end{aligned}$$

From (6), (7) and (13), we infer

14 $$\begin{aligned} p+q+pq<0. \end{aligned}$$

Let $\lambda$ denote the multiplier of the cycle $\{p, q\}$. We have

$$\begin{aligned} \lambda =f'_\mu (p)\cdot f'_\mu (q) =(p+1)\exp (p+\mu )\cdot (q+1)\exp (q+\mu ). \end{aligned}$$

It follows from (12) that

15 $$\begin{aligned} \lambda =(p+1)(q+1)=p+q+pq+1. \end{aligned}$$

Hence by (14), we get $\lambda <1$. Furthermore, we have the following result.

### **Lemma 15**

*The multiplier*$\lambda$*as a function of*$\mu$*defined in*$(2,+\infty )$*is decreasing, and its range is*$(-\infty , 1)$.

### *Proof*

From (12), we have

16 $$\begin{aligned} \frac{dp}{d\mu }+\frac{dq}{d\mu }+2=0. \end{aligned}$$

Moreover, since $q=f_\mu (p)=p\exp (p+\mu )$, i.e., $\log (q/p)=p+\mu$, we have

17 $$\begin{aligned} \frac{1}{q}\cdot \frac{dq}{d\mu }-\frac{1}{p}\cdot \frac{dp}{d\mu }=\frac{dp}{d\mu }+1. \end{aligned}$$

From (16) and (17), by direct calculation, we obtain

18 $$\begin{aligned} \frac{dp}{d\mu }=-\frac{p(q+2)}{p+q+pq} \end{aligned}$$

and

19 $$\begin{aligned} \frac{dq}{d\mu }=-\frac{q(p+2)}{p+q+pq}. \end{aligned}$$

From (15), we have

$$\begin{aligned} \frac{d\lambda }{d\mu }=\frac{dp}{d\mu }+\frac{dq}{d\mu }+q\frac{dp}{d\mu }+p\frac{dq}{d\mu }. \end{aligned}$$

Then by (16), (18) and (19), we get

20 $$\begin{aligned} \frac{d\lambda }{d\mu }=-2-\frac{pq(p+q+4)}{p+q+pq}. \end{aligned}$$

By (12) and $\mu >2$, we have $p+q+4=-2\mu +4<2$. Hence by (6), (14) and (20), we infer $\frac{d\lambda }{d\mu }<0$, thus $\lambda$ as a function of $\mu$ is decreasing.

Following (6), (14) and (18), we get $\frac{dp}{d\mu }<0$, hence *p* as a function of $\mu$ is decreasing, and then $\lim _{\mu \rightarrow {2^{+}}}p$ exists, say $p_0$. Then from $g_\mu (p)=0$, we have $g_2(p_0)=0$. Since the function $g_2(x)$ is increasing in $(-\infty , +\infty )$, we get $p_0=-2$. Hence from (12) and (15), we have

$$\begin{aligned} \lim _{\mu \rightarrow {2^{+}}}q=\lim _{\mu \rightarrow {2^{+}}}(-p-2\mu )=-2 \end{aligned}$$

and

21 $$\begin{aligned} \lim _{\mu \rightarrow {2^{+}}}\lambda =\lim _{\mu \rightarrow {2^{+}}}(p+1)(q+1)=1. \end{aligned}$$

By (6) and (12), we have

$$\begin{aligned} |q|=|p\exp (p+\mu )|=\frac{|q+2\mu |}{\exp (q+\mu )}<\frac{2+2\mu }{\exp (\mu -2)}. \end{aligned}$$

It is easy to obtain by calculating that

$$\begin{aligned} \lim _{\mu \rightarrow {+\infty }}\frac{2+2\mu }{\exp (\mu -2)}=0, \end{aligned}$$

which implies $\lim _{\mu \rightarrow {+\infty }}q=0.$ Hence from (12) and (15), we have

22 $$\begin{aligned} \lim _{\mu \rightarrow {+\infty }}\lambda =\lim _{\mu \rightarrow {+\infty }}(-q-2\mu +1)(q+1)=-\infty . \end{aligned}$$

Since $\lambda$ is decreasing, by (21) and (22), we obtain that the range of $\lambda$ is $(-\infty , 1)$. $\square$

**Proof of Theorem**3

### *Proof*

Set $\mu _{*}:=\lambda ^{-1}(-1)$. Then by Lemma 15, we have $\mu _{*}\ne +\infty$ and $\lambda ^{-1}(-1,1)=(2, \mu _{*})$. By Theorem 13 and Lemma 14, we deduce that $\mu \in B_2$ if and only if $\mu >2$ and $\lambda (\mu )\in (-1,1)$. Hence we obtain $B_2=(2, \mu _{*})$.

Therefore, Theorem 3 is proved completely. $\square$

### *Remark 16*

Computation of $$ \mu _*$$.

From (12) and (15), we have

$$\begin{aligned} \lambda =(1-2\mu -q)(1+q)\ \ \ \Leftrightarrow \ \ \ (q+\mu )^2=(\mu -1)^2-\lambda . \end{aligned}$$

Noting $q\in (-2, 0)$ and $\mu >2$, we obtain

$$\begin{aligned} q=\sqrt{(\mu -1)^2-\lambda }-\mu , \end{aligned}$$

and then

$$\begin{aligned} p=-\sqrt{(\mu -1)^2-\lambda }-\mu . \end{aligned}$$

Hence the equation $p=q\exp (q+\mu )$ with $\lambda (\mu _*)=-1$ implies that $\mu _*$ is the root of the function

$$\begin{aligned} \Phi (\mu )=\frac{w+\mu }{w-\mu }+\exp (w)\ \ \text{ with }\ \ w:=\sqrt{(\mu -1)^2+1}. \end{aligned}$$

**Fig. 1:**
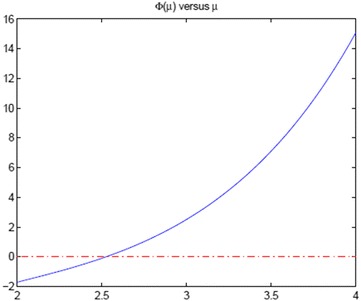
$$\Phi (\mu ), \ 2\le \mu \le 4$$

The plot of $\Phi (\mu )$ is as in Fig. 1. One can compute the root of $\Phi (\mu )$ up to machine precision with numerical methods like bisection, secant method and so on.

*Result*: $$ \mu _*= 2.526467725\cdots $$

(with $$ p = -4.351324903\cdots $$ and $$ q = -0.701610548\cdots $$).

### *Remark 17*

The Taylor series expansion of $\lambda (\mu )$.

From (12), (15) and (20), we have

$$\begin{aligned} \frac{d\lambda }{d\mu }&=-2-\frac{pq(p+q+4)}{p+q+pq}\\&=-2-\frac{(2\mu +\lambda -1)(4-2\mu )}{\lambda -1}\\&=\frac{2(\lambda -1)((\mu -2)-1)+4(\mu -2)^2+8(\mu -2)}{\lambda -1}, \end{aligned}$$

which implies that

23 $$\begin{aligned} (\lambda -1)\frac{d\lambda }{d\mu } =2(\lambda -1)((\mu -2)-1)+4(\mu -2)^2+8(\mu -2). \end{aligned}$$

Suppose that the formal Taylor series expansion with expansion point 2 is

$$\begin{aligned} \lambda (\mu )=\sum ^{\infty }_{k=0}a_k(\mu -2)^k, \end{aligned}$$

then

$$\begin{aligned} \frac{d\lambda (\mu )}{d\mu }=\sum ^{\infty }_{k=1}ka_k(\mu -2)^{k-1}. \end{aligned}$$

Note that $a_0=\lambda (2^+)=1$, and let $t:=(\mu -2)$, from (23) we have

$$\begin{aligned} \left( \sum ^{\infty }_{k=1}a_{k}t^k\right) \left( \sum ^{\infty }_{k=1}ka_{k}t^{k-1}\right) =2\sum ^{\infty }_{k=1}a_{k}t^{k+1}-2\sum ^{\infty }_{k=1}a_{k}t^{k}+4t^2+8t, \end{aligned}$$

which implies that

$$\begin{aligned} \sum ^{\infty }_{l=1}\left( \sum ^{l}_{j=1}ja_{j}a_{l+1-j}\right) t^{l} =t(8-2a_1)+t^2(2a_1-2a_2+4)+\sum ^{\infty }_{l=3}2(a_{l-1}-a_l)t^{l}. \end{aligned}$$

The comparison of the left and the right side with respect to $t^l$ yields

$$\begin{aligned}&l=1:\ \ \ a_1^2=8-2a_1\ \Rightarrow a_1=-1\pm 3\ \Rightarrow \frac{d\lambda (\mu )}{d\mu )}|_{\mu =2}=a_1=-4<0;\\&l=2:\ \ \ a_1a_2+2a_2a_1=2a_1-2a_2+4\ \Rightarrow a_2=0.4;\\&l\ge 3:\ \ \ a_l=\frac{1}{4l+2}\left( \sum ^{l-1}_{j=2}ja_{j}a_{l+1-j}-2a_{l-1}\right) . \end{aligned}$$

**Fig. 2:**
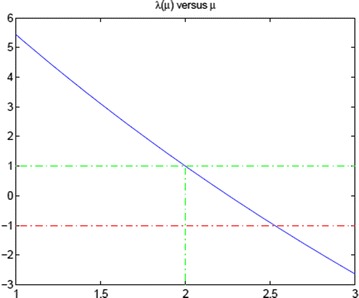
Taylor series expansion of $\lambda (\mu )$

The Plot of the Taylor series expansion of $\lambda (\mu )$ up to 10th order with expansion point 2 is as in Fig. 2. Indeed, the Taylor polynomials can also be used to approximate $\mu _*$.

## The Proof of Theorem 4

In this section we prove Theorem 4 by finding a parameter $\mu _n$ such that $f_{\mu _n}$ has super-attracting periodic points of *n*-order. This parameter $\mu _n$ satisfies the equation $f_{\mu }^{n}(-1)=-1$, i.e., $\mu$ belongs to the set

$$\begin{aligned} E_n:=\{\mu \in {\mathbb {R}} | s_n(\mu )=-1\}, \end{aligned}$$

where $s_n(\mu ):=f_{\mu }^n(-1)$ for every positive integer *n*.

### **Lemma 18**

(Jang (1992)) *Let*$n\ge 2$, *then*$s_n(\mu )\rightarrow 0$*as*$\mu \rightarrow +\infty$.

### **Lemma 19**

*For every positive integern* , $E_n$*is a finite set, and*$E_1=\{1\}\subset E_n$.

### *Proof*

Since $s_1(\mu )=f_{\mu }(-1)=-\exp (\mu -1)$, we get $E_1=\{1\}$, and from $s_n(1)=-1$, we get $\{1\}\subset E_n$.

Now, suppose $n\ge 2$. Since $s_1(\mu )=f_\mu (-1)$ is the minimum value of $f_\mu$, we have

$$\begin{aligned} s_n(\mu )\ge f_\mu (-1)=-\exp (\mu -1)>-1, \ \ \ \text{ for } \text{ every }\ \ \mu <1. \end{aligned}$$

Hence $\mu \ge 1$ for every $\mu \in E_n$. From Lemma 18, there is $M_n$ such that $s_n(\mu )>-1$ for every $\mu >M_n$. Hence $\mu \le M_n$ for every $\mu \in E_n$. Thus $E_n$ is a bounded set, and then is a finite set. $\square$

Lemma 19 allows us to define $\mu _n:=\max \{\mu \in E_n\}$. Clearly, $\mu _1=1$ and $\mu _n\ge 1$ for every $n\ge 2$.

### **Lemma 20**

*Let*$n\ge 2$. *If*$\mu >\mu _n$, *then*$s_n(\mu )>-1$*and*$s_{n+1}(\mu )<s_{n}(\mu )$.

### *Proof*

It is easy to see that $s_n(\mu )<0$ for every $\mu$. Set

$$\begin{aligned} I_n:=\{s_n(\mu ) | \mu \in (\mu _n,+\infty )\}. \end{aligned}$$

Clearly, $I_n$ is an interval, and $I_n\subset (-\infty , 0)$. Note that $-1\not \in I_n$ from the definition of $\mu _n$. Hence by Lemma 18 and $s_n(\mu _n)=-1$, we infer $I_n=(-1,0)$. Hence if $\mu >\mu _n$, then $-1<s_n (\mu )<0$. Noting $\mu _n\ge 1$, we have

$$\begin{aligned} s_{n+1}(\mu )= s_{n}(\mu )\exp (s_{n}(\mu )+\mu )< s_{n}(\mu )\exp (\mu _n-1)\le s_{n}(\mu ). \end{aligned}$$

Thus we obtain the assertion. $\square$

The following two Lemmas have been proved by Kuroda and Jang (1997). Here we give different proofs of them.

### **Lemma 21**

*The sequence*$\{\mu _n\}_{n=1}^{\infty }$*is increasing.*

### *Proof*

Since $\mu _n\ge 1$ and $s_{n}(\mu _{n})=-1$ for every $n\ge 1$, we have

$$\begin{aligned} s_{n+1}(\mu _{n})= s_{n}(\mu _{n})\exp (s_{n}(\mu _{n})+\mu _n)=-\exp (\mu _n-1)\le -1. \end{aligned}$$

Since

$$\begin{aligned} \frac{d s_{n+1}}{d \mu }=((1+ s_{n})\frac{d s_n}{d \mu }+ s_{n}) \exp (s_{n}+\mu ), \end{aligned}$$

we get

$$\begin{aligned} \frac{d s_{n+1}}{d \mu }|_{\mu =\mu _n} =-\exp (\mu _n-1) < 0. \end{aligned}$$

Hence there exists $\mu _{n}'> \mu _{n}$ such that $s_{n+1}(\mu _{n}') < s_{n+1}(\mu _{n}) \le -1$. Following Lemma 20, we deduce $\mu _{n}' < \mu _{n+1}$, and then $\mu _{n} < \mu _{n+1}$. Thus $\{\mu _n\}_{n=1}^{\infty }$ is increasing. $\square$

### **Lemma 22**

*For every positive integern* , *the function*$f_{\mu _n}$*has super-attracting periodic points of n*-*order.*

### *Proof*

Clearly, $f_{\mu _1}$ has the super-attracting fixed point $-1$. Since $\mu _2>1$, we have

$$\begin{aligned} s_1(\mu _2)=-\exp (\mu _2-1)<-1 = s_2(\mu _2). \end{aligned}$$

This implies that $s_1(\mu _2), s_2(\mu _2)$ are super-attracting periodic points of 2-order of $f_{\mu _2}$.

Let $n\ge 3$ and $k=2,3,\ldots ,n-1$. Then $\mu _n>\mu _k>1$ from Lemma 21. Hence by Lemma 20, we have

$$\begin{aligned} s_1(\mu _n)=-\exp (\mu _n-1)<-1 = s_n(\mu _n)<s_{n-1}(\mu _n)< \cdots < s_2(\mu _n). \end{aligned}$$

This implies that $s_1(\mu _n), s_2(\mu _n), \cdots , s_{n}(\mu _n)$ are super-attracting periodic points of *n*-order of $f_{\mu _n}$. $\square$

From Lemma 22, we immediately get the following Theorem.

### **Theorem 23**

*For every positive integern* , $\mu _n\in {B_n}$.

Thus, Theorem 4 follows from Theorem 23.

## The Proof of Theorem 5

Let $\hat{B}_n$ be the set of real parameters $\mu$ such that $f_{\mu }$ has a periodic point of *n*-order, whose multiplier is less than 1. It follows from the implicit function theorem that the set $\hat{B}_{n}$ is an open set for every positive integer *n*.

### **Theorem 24**

*Suppose thatpis a prime number,* (*a*, *b*) *is a component of*$\hat{B}_p$. *Then*$f_a$*has parabolic periodic points ofp*-*order. Furthermore,*$a\in \partial {B_p}$.

### *Proof*

From Theorem 13, we know that if $\mu \le 2$, then $f_\mu$ has no periodic points of *p*-order. Hence we have $a\ge 2$. Choose a decreasing sequence $\{a_n\}_{n=1}^{\infty }\subset (a,b)$ such that $\lim _{n\rightarrow \infty }a_n=a$, and choose a periodic point $x_n$ of *p*-order of $f_{a_n}$ with multiplier $\lambda _n<1$. From Claim 2 in “Dynamics of *f*_*μ*_(*x*) for *μ* ≤ 2 and the Proof of Theorem 3” section, we have $x_n <0$. Since $s_1(\mu )$ is the minimum value of $f_\mu$ and $s_1(\mu )=-\exp (\mu -1)$ as a function of $\mu$ is decreasing, we have

$$\begin{aligned} x_n\in [s_1(a_n),0]\subset {[s_1(a_1),0]}. \end{aligned}$$

Thus we may suppose that $\{x_n\}_{n=1}^{\infty }$ is a convergent sequence, otherwise we consider a subsequence of $\{x_n\}_{n=1}^{\infty }$. Set $x_0:=\lim _{n\rightarrow \infty }x_n$, then we have

$$\begin{aligned} f_a^p(x_0)=\lim _{n\rightarrow \infty }f_{a_n}^p(x_n)= \lim _{n\rightarrow \infty }x_n= x_0 \end{aligned}$$

and

$$\begin{aligned} \lambda _0:= \lim _{n\rightarrow \infty }\lambda _n= \lim _{n\rightarrow \infty }(f_{a_n}^p)'(x_n)= (f_{a}^p)'(x_0). \end{aligned}$$

Hence $x_0$ is a fixed point of $f_a^p$, and its multiplier $\lambda _0\le 1$ from $\lambda _n<1$. Since *p* is a prime number, $x_0$ is either fixed point or periodic point of *p*-order of $f_a$.

Assume $x_0$ is not a periodic point of *p*-order of $f_a$, then $x_0$ is a fixed point of $f_a$. Thus $x_0=0$ or $x_0=-a$. Noting $(f_a^p)^\prime (0)=e^{pa}>1$, we have $x_0=-a$. Define the function

$$\begin{aligned} F(x,\mu ):=f_\mu ^p(x)-x,\ \ \ (x,\mu )\in \mathbb {R}^2. \end{aligned}$$

We have

$$\begin{aligned} F_x^\prime (x_0,a)=(f_a^p)^\prime (x_0)-1=(1-a)^p-1<0. \end{aligned}$$

Hence there exists a disc *D* which is a neighborhood of $(x_0,a)$ such that $F_x^\prime (x,\mu )<0$ for every $(x,\mu )\in D$. Choose a positive integer *m* such that $(x_m,a_m)\in D$ and $(-a_m,a_m)\in D$. Note that $x_m\ne -a_m$, and $F(x_m,a_m)=0, F(-a_m,a_m)=0$. Then by Rolle theorem, there exists a point $(y_0,a_m)\in D$ such that $F_x^\prime (y_0,a_m)= 0$. It contradicts that $F_x^\prime (y_0,a_m)< 0$. Hence $x_0$ is a periodic point of *p*-order of $f_a$.

Because of $a\notin \hat{B}_p$, we infer that $\lambda _0=1$. Hence $x_0$ is a parabolic periodic point of *p*-order of $f_a$. Since $\lambda _0= \lim _{n\rightarrow \infty }\lambda _n$, we infer that $a_n\in {B_p}$ for large enough *n*. This implies $a\in \partial {B_p}$. Hence, Theorem 24 is completed. $\square$

The proof of Theorem 5 needs Theorem 24 and the following Lemmas.

### **Lemma 25**

(Li and York 1975, Li-York theorem) *LetIbe a closed interval, and*$f: I \rightarrow I$*be a continuous mapping. Iffhas periodic points of 3-order, thenfhas periodic points ofn* -*order for every positive integern*.

### **Lemma 26**

(Deng and Cai (1993)) *Let*$f: {\mathbb {R}} \rightarrow {\mathbb {R}}$*be a continuously differentiable function. Suppose thatfhas two fixed points*$x_1, x_2$, *say*$x_1<x_2$. *If their multipliers are greater than 1, thenfhas another fixed point*$x_3\in (x_1,x_2)$.

**Proof of Theorem**5

### *Proof*

By assumption, the prime number $p\ge 5$.

Set $I_0:=[s_1(\mu _3),0]$ and $g: =f_{\mu _3}|I_0$. Since $s_1(\mu _3)$ is the minimum value of $f_{\mu _3}$, we see that *g* is a self-mapping of $I_0$. From Lemma 22, *g* has super-attracting periodic points of 3-order. According to Lemma 25, *g* has periodic points of *p*-order. Since the Fatou set $F(f_\mu )$ has at most one cycle of periodic components, we get that all of the periodic points of *p*-order of *g* are repelling. Let $n_p$ denote the number of the cycles of periodic points of *p*-order of *g* , and $n'_p$ (resp. $n''_p$) denote the number of the cycles of which the multipliers are greater (resp. less) than 1. Clearly, $1 \le n_p <+\infty$. Since the periodic points of *p*-order of *g* are repelling, we have $n'_p + n''_p = n_p$. Since *p* is a prime number, every fixed point of $g^{p}$ is either fixed point or periodic point of *p*-order of *g*. Noting that *g* has only two fixed points 0 and $-\mu$, we get that $g^p_\mu$ has $pn_p + 2$ fixed points. Assume $n''_p=0$, then $g^p_\mu$ has at least $pn_p$ fixed points, whose multipliers are greater than 1. However, by Lemma 26, we deduce that $g^p_\mu$ has at least $2pn_p-1$ fixed points, and then $pn_p + 2\ge 2pn_p-1$, which contradicts that $pn_p\ge p \ge 5$. Hence we obtain $n''_p\ne 0$, which implies $\mu _3\in \hat{B}_{p}$.

Let (*a*, *b*) be the component of $\hat{B}_{p}$, which contains $\mu _3$. Then by Theorem 24, there exists $a_0$ such that $a_0<\mu _3$ and $a_0\in {B_p}$. From Lemma 21, $\mu _3<\mu _p$. Since $\mu _3\notin {B_p}$, we infer that there exist two different components of $B_p$, one of them contains $a_0$, the other contains $\mu _p$.

Thus, Theorem 5 is proved completely. $\square$

## Conclusions

It is known that the dynamics given by the iteration of transcendental entire maps has been widely studied. In this paper, we consider the dynamics of the functions $f_{\mu }(z)=ze^{z+\mu }$ with the real parameter, and prove that the Fatou set $F({f_{\mu }})$ is a completely invariant attracting basin for every parameter $\mu <0$. We say that a real parameter $\mu$ belongs to the set $B_n$ for a positive integer *n* if $f_{\mu }$ has an attracting cycle of *n*-order. Regarding the set $B_n$ for $n>1$, we show that (1) there exists $\mu _*\ne +\infty$ such that $B_2=(2,\mu _*)$; (2) for every positive integer $n>2$, the set $B_n$ is non-empty; (3) for every prime number $p>3$, the set $B_p$ has at least two components.
